# Image reconstruction from photon sparse data

**DOI:** 10.1038/srep42164

**Published:** 2017-02-07

**Authors:** Lena Mertens, Matthias Sonnleitner, Jonathan Leach, Megan Agnew, Miles J. Padgett

**Affiliations:** 1 School of Physics and Astronomy, University of Glasgow, Glasgow, G12 8QQ, UK; 2 Department of Physics, Heriot-Watt University, Edinburgh, EH14 4AS, UK

## Abstract

We report an algorithm for reconstructing images when the average number of photons recorded per pixel is of order unity, i.e. photon-sparse data. The image optimisation algorithm minimises a cost function incorporating both a Poissonian log-likelihood term based on the deviation of the reconstructed image from the measured data and a regularization-term based upon the sum of the moduli of the second spatial derivatives of the reconstructed image pixel intensities. The balance between these two terms is set by a bootstrapping technique where the target value of the log-likelihood term is deduced from a smoothed version of the original data. When compared to the original data, the processed images exhibit lower residuals with respect to the true object. We use photon-sparse data from two different experimental systems, one system based on a single-photon, avalanche photo-diode array and the other system on a time-gated, intensified camera. However, this same processing technique could most likely be applied to any low photon-number image irrespective of how the data is collected.

Traditional camera systems produce images based on many tens of thousands of detected photons per pixel^1^. However, the desire for low-light imaging coupled with the availability of modern sensor technologies means there is both desire and methods for imaging where the number of detected photons per pixel is of order unity, i.e. photon-sparse. The resulting photon-number data is inherently of an integer nature and subject to Poissonian statistics around a fractional expectation value; the true pixel intensity. Hence, at these low photon-numbers, even an object of uniform intensity gives a noisy image^2^. Beyond the data itself, taking into account both the dark-count rate of the pixel and assumptions about the object improves the correct reconstruction of the true pixel intensities.

In this work we take integer photon data produced using cameras able to detect individual photons, and reconstruct grey-scale images. The image optimization uses the minimisation of a cost function that incorporates both a log-likelihood term based on measured data and regularization term based upon the sum of the moduli of the second spatial derivatives of the image pixel intensities (total curvature of the image). The desired balance between these two terms gives the most regularised image that is statistically consistent with the data. Previous techniques for such images have relied on similar approaches^3^,^4^,^5^.

We begin this paper by introducing the log-likelihood and total curvature of an image and use these quantities to define a cost function for the reconstruction. We also describe a bootstrapping technique to calculate a suitable balance between the terms within this cost function. When compared to the recorded data, our processed images exhibit lower residuals with respect to the true image. Such processing techniques could apply to any low photon-number image and impact many photon-sparse imaging systems.

## Log-Likelihood Of A Proposed Image Given The Measured Data

When considering the degree to which a proposed solution fits the *N* measured data points, it is common practice to assume a Gaussian noise term and calculate a metric based upon Pearson’s chi-squared test^6^ which is anticipated to have a value of order unity. However, for low photon numbers where the data is both positive and integer, a Gaussian noise model is no longer appropriate. As used elsewhere^7^, a better choice of noise model for this sparse data is a Poissonian distribution^6^,^8^.

If the number of photons recorded by the *j*^*th*^ pixel is *n*_*j*_, we wish to establish the log-likelihood that the true pixel intensity is given by *I*_*j*_. For an anticipated dark level of *ε* fractional counts per pixel, the probability distribution of *n*_*j*_ given *I*_*j*_ is


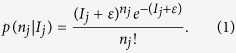


The log-likelihood^9^ is a quantity derived from the above distribution and is a measure of consistency between a reconstructed image and the given data. This log-likelihood for the whole image is defined over the sum of *N* individual pixels as:


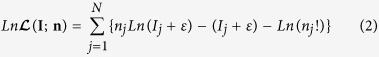


in which *I*_*j*_ is the fractional intensity of each pixel in the reconstructed image, *n*_*j*_, is the photon count of each pixel (i.e. the measured data) and *ε* is the estimated dark-count rate per pixel (typically a small number). See Appendix A.

## Defining A Cost Function For The Image Reconstruction

Images of real objects tend to exhibit strong correlations between neighbouring pixels, or equivalently are sparse in their spatial frequencies. It is convenient to take this desire for smoothness, expressed as a function *R(I*_*j*_), and combine it with the log-likelihood term to create a single cost function. The optimum image reconstruction is then simply that which gives the lowest overall cost


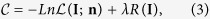


where *λ* is known as the Regularisation factor and is a choice of positive floating point number. It simply defines the balance between the two terms and determines how ‘smooth’ (with respect to *R*) the image becomes through the optimization process.

A number of different functions may be used as a regularisation term. As discussed above we choose the total curvature (TC) of an image as defined in Equation (4) and we also use the total of the curvature squares (*TC*^2^, 5). This use of a total curvature regulariser is consistent with a Bayesian model under the assumption that the second spatial derivative follows a normal distribution.


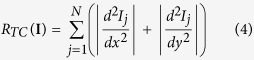



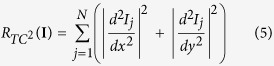


Our algorithm, which is discussed below, changes individual pixels at random and recalculates the cost function of the resulting image to determine whether that random change improved the image, so in particular it must calculate the chosen regularisation function after each such change. For Total Curvature based functions, this is calculated locally around the pixel that was altered; and so these are considerably faster than an often used spatial-frequency participation function^9^, which needs to recalculate the Discrete Cosine Transform over the whole image every time a change is made. Indeed, Total Variation or Curvature based algorithms are also widely used to suppress noise in conventional image processing^10^,^11^.

## Monte Carlo Optimization Of Cost Function And Image Reconstruction

**Figure 1:**
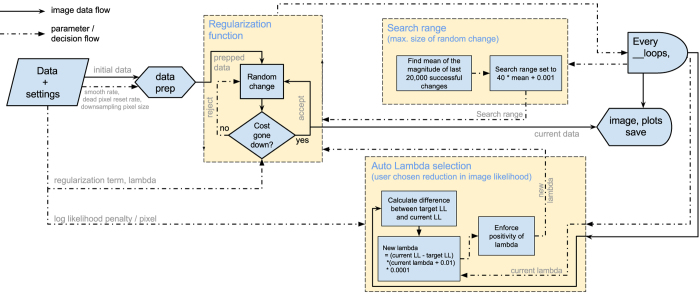
Process flow diagram for the optimization algorithm. The cost and log-likelihood (LL) of the input image is calculated. A pixel is then chosen at random, to be altered by a random amount (typically ± *m*, where *m* is a floating point number), and the cost is calculated again. If the cost has decreased, the change is accepted. If not, it is rejected. A new pixel is chosen and altered, and this process is repeated thousands of times until, eventually, a better image is formed. This is a stochastic process, and running the algorithm more than once with the same image and input parameters may yield slightly different (though cosmetically similar) results.

Having established a cost function for the image reconstruction, a variety of possible algorithms exist to find the reconstruction having minimum cost. In this work we use a Monte-Carlo approach where pixels selected at random from within the image are subject to a random intensity change (whilst enforcing positivity). After each iteration, the cost function is re-evaluated and if the result is a lower overall cost then the change is kept whereas if the cost is higher then the change is discarded. Once the cost function stabilises we assume that the image reconstruction is optimized. A flow diagram for this algorithm is shown in Fig. 1.

Of course, more sophisticated algorithms for optimisation of solutions giving lowest cost are available^12^. However, within this work we have chosen to favour the robustness of the Monte-Carlo approach in finding the global minimum of the cost function over the potential higher speed of alternative approaches. Having established the efficacy of this particular cost function, reducing the time taken for convergence of the image through more efficient optimisation algorithms and/or graphics card based processing (which have no impact on the solution itself) could be the subject of further work.

Within our Monte-Carlo approach a number of other background processes improve the speed at which the algorithm converges upon an optimised image; these processes include rescaling of the ‘search range’ (the numeric range from which the change added to altered pixels is chosen, this is rescaled based on the magnitudes of the previous 20,000 successful changes), and an optimization loop for the regularization parameter to reach a target value of the log-likelihood.

## Strategies For Setting The Degree Of Regularisation With The Optimisation, i.e. *λ*-Selection

One approach to the optimisation of the regularisation factor is user selection between the various images obtained for different values of *λ*. However, this reliance on the user is somewhat arbitrary and subject to user bias.

In situations where the noise inherent in the system is well understood and quantified, other deterministic approaches for setting *λ* can be adopted. In essence, *λ* can be increased until the reconstructed image deviates from the original data only by an extent compatible with the anticipated measurement noise. If the noise associated with each of *N* measurements is Gaussian in nature then this deviation from the original data corresponds to *χ*^2^/*N* ≈ 1 in Pearson’s chi-squared test (this is equivalent to the data differing from the fit by an average amount corresponding to the noise in the measurement). For Poissonian data, the expected deviation of the optimized image from the original data is more complicated (see Appendix B). When the number of photons recorded per pixel, *n*_*j*_, is high then the Poissonian distribution tends to a Gaussian distribution with a standard deviation of 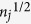.

In the absence of any additional information, for a given *n*_*j*_, the most likely value of *I*_*j*_ is itself *n*_*j*_. Reducing or increasing *I*_*j*_ by 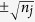 gives a reduction in the log-likelihood of 0.5 per image pixel, which when summed over the entire image give a target reduction in the log-likelihood of 0.5 *N*.

**Figure 2:**
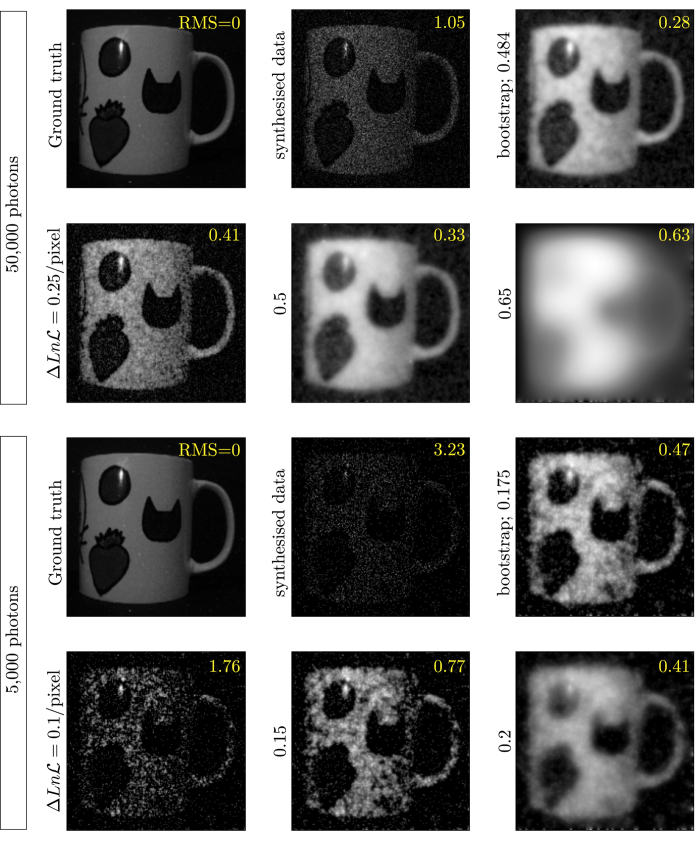
Synthesised input images and optimization results, using Total Curvature-squared as the regularization term within the cost function. We are using the Root Mean Square of each image against the ground truth to determine the quality of each reconstruction. The two data sets shown contain 50,000 and 5,000 photons. The first row of each set shows the ground truth, the respective synthesised data set and the reconstruction obtained using bootstrapping (with log-likelihood/pixel penalties of 0.484 and 0.175 for the 50,000 and 5,000-photon images, respectively), while the second row shows three further reconstructions with different log-likelihood/pixel (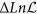) penalties for comparison (0.25, 0.5, 0.65 and 0.1, 0.15, 0.2 for the 50,000 and 5,000-photon images, respectively). The Root Mean Square normalised with respect to the average image intensity (RMS, inset) is shown for each image. Image sizes are all 240 × 240 pixels.

Figure 2 shows an image of a mug and two synthesized 240 × 240 pixel data sets corresponding to 50,000 and 5,000 photons and various reconstructed images for different reductions in log-likelihood


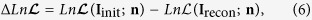


with the initial intensity **I**_init_ = n and the reconstructed image **I**_recon_.

For each of these images we calculate the root mean square deviation of the reconstructed image with respect to the known original. In the case of high photon-number, we can anticipate that the image for which the reduction in log-likelihood gives 0.5/pixel is the image corresponding to the maximum statistically allowed value of *λ*. It is therefore reassuring to note that the images reconstructed under this constraint appear to correspond to the value of *λ* which gives a reconstruction with the smallest Root Mean Square (RMS) intensity error with respect to the true image. However, as mentioned above, this 0.5/pixel assumption regarding the optimum reductions in log-likelihood is not valid for small photon numbers.

To find an appropriate reduction in log-likelihood we adopt a bootstrapping technique in which we apply a mild low-pass smoothing filter to the raw data to give a first approximation to the true image. This approximation image is then used as a probability distribution from which to synthesize mock data containing the same number of photons as the measured data set. Taking these mock data sets and comparing them to the approximation image we calculate the corresponding log-likelihood values of the approximation image given the mock data. Averaging these log-likelihoods together over the various mock data give us a target log-likelihood for reconstructing an image based on the measured data.

**Figure 3:**
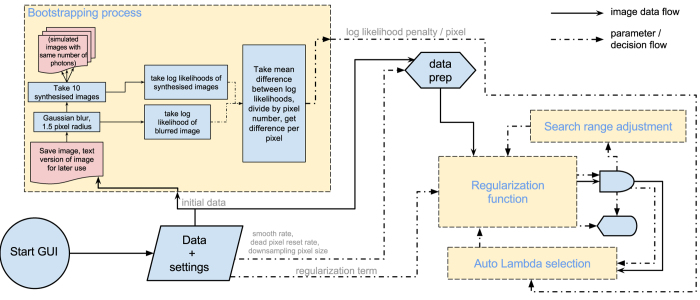
Program flow diagram showing the bootstrapping process, omitting details of the others (which are shown in Fig. 1).

Having established the target log-likelihood for a specific data set we set this value as a target for the reconstruction. Using the original raw data we reconstruct our final image that minimizes the cost function, with an optimization of *λ* to give this target value of log-likelihood. A flow diagram for this algorithm is shown in Fig. 3.

**Figure 4:**
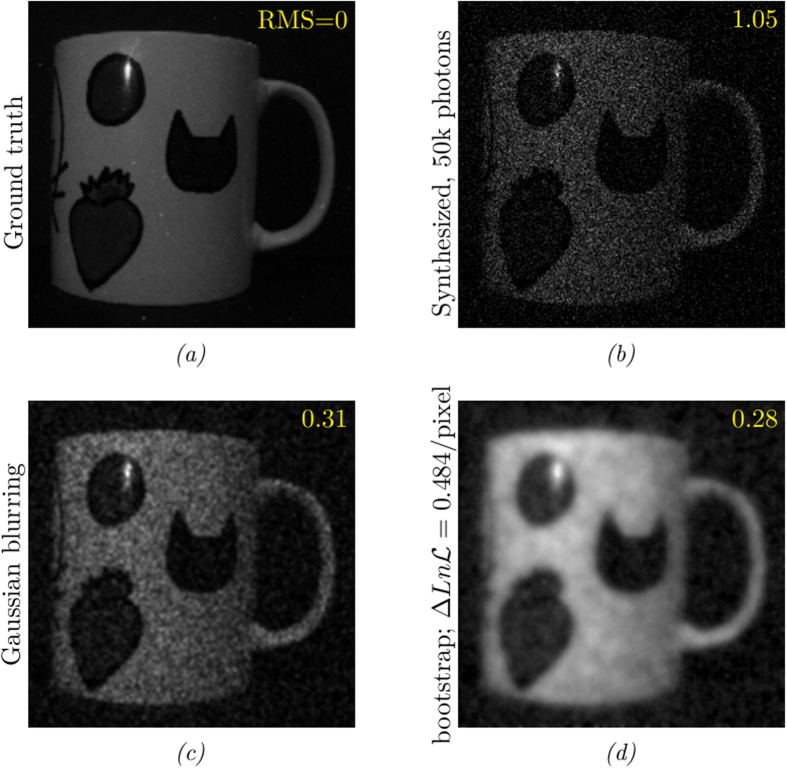
Regularization, using the bootstrapping method to choose the log-likelihood penalty. (**a**) ground truth image (**b**) synthesised data with 50,000 photons (**c**) Gaussian-smoothed image with a pixel radius of 1.5 (**d**) reconstructed image using bootstrapping, optimizing Total Curvature with squared terms. Bootstrapping gives a better RMS residual than the naively smoothed image.

Figure 4 shows a test image of the same mug, but with a synthesized data set corresponding to 50,000 photons and the reconstructed image whilst setting the target log-likelihood for the reconstruction following the bootstrapping procedure described above. Again we see that the optimised value of *λ* corresponding to the target value of log-likelihood gives the reconstructed image with the lowest value of RMS intensity error compared to the true image. Perhaps more importantly the reconstructed image is better than the spatially filtered image upon which the bootstrapping of the target log-likihood was itself based. Note that this optimisation procedure requires no user set parameters, rather it is the data itself from which the optimum degree of regularisation is determined.

## Image Optimization Algorithm Applied To Experimental Systems

Taking the algorithm described above we apply it to the reconstruction of images from photon-sparse data as obtained by the imaging system described below.

The mug data was obtained using a single-photon, avalanche detector (SPAD) camera where each individual pixel is a single-photon sensitive detector that provides a binary response to a photon input within a user defined gate time, which in this work was set to 100 *μ*s. This time-gated SPAD cameras had a spatial resolution of 320 × 240 pixels (from which we extract a 240 × 240 region), a fill factor of 26%, a minimum exposure time of ≈10 ns, and maximum a frame rate of approximately 10 kHz^13^. The true grayscale image on which the calculation of the RMS error was based was obtained by adding multiple binary images together. In all of the images, we removed the count value of any pixels with dark count rates greater than 1000 counts per second, replacing them with the average of the surrounding pixels.

**Figure 5:**
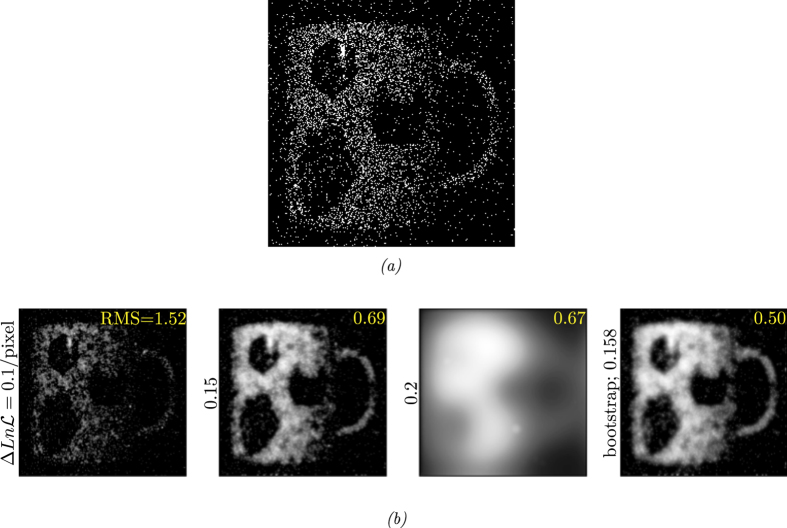
Original data (Root Mean Square = 3 .**02 compared with ground truth from** Fig. 2).

Figure 5 shows an image obtained from this time-gated SPAD array^14^ where the photon count in each pixel of the image is either 1 or 0. From the quality of the reconstructed images in Fig. 5b we see that the bootstrapping technique defines a target value of the log-likelihood that results in an appropriate value of *λ* giving reconstructed images which appear to be a good balance between the measured data and the assumptions of image smoothness.

**Figure 6:**
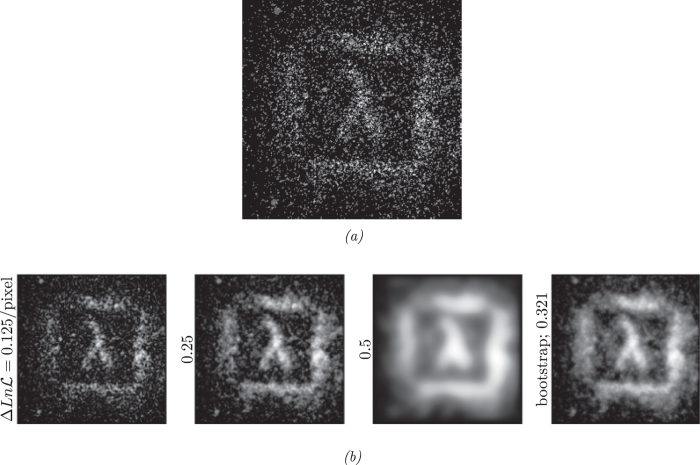
Time-gated ghost imaging system data and its reconstructions using our optimization algorithm.

Figure 6 shows a further example of experimentally obtained data, but this time from a time gated ghost imaging system as fully described in ref. 15. The reconstructions are shown in Fig. 6b. In this last case no true image was obtainable and hence it was not possible to calculate the RMS pixel error, however, again the bootstrapping techniques appears to give a near optimum balance between the measured data and assumptions about image smoothing.

## Conclusion

This paper presents a numerical optimization algorithm for reconstructing photon-sparse images (i.e. data where the average number of photons recorded per pixel is of order unity). The optimization was based on the minimisation of a cost function which combined a statistical measure of how well the reconstructed image matched the recorded data with an additional constraint regarding image smoothness. Rather than relying on user selection to determine the optimum balance between data and smoothing, we used a bootstrapping technique to derive a target value of the log-likelihood based upon an initial image obtained from low-pass filtering. This algorithm (with bootstrapping) was tested on both simulated and real data, in both cases giving reconstructions with an appropriate level of image smoothness and consistency with the recorded data.

It was also apparent that the resulting reconstructions were better than those based upon low-pass spatial filtering and the bootstrapping technique could replace user intervention. This image processing algorithm can be applied to the data obtained from any imaging system in which the number of measured photons per image pixel is small.

## Additional Information

**How to cite this article**: Mertens, L. *et al*. Image reconstruction from photon sparse data. *Sci. Rep.* **7**, 42164; doi: 10.1038/srep42164 (2017).

**Publisher's note:** Springer Nature remains neutral with regard to jurisdictional claims in published maps and institutional affiliations.

## Supplementary Material

Supplementary Appendix A, B
