# Supplementary material for: Image reconstruction from photon sparse data
Source: Sci Rep. 2017 Feb 7;7:42164. doi: 10.1038/srep42164 (PMC5294572; doi:10.1038/srep42164)
Supplement: Supplementary Appendix A, B [file srep42164-s1.pdf]

Image reconstruction from photon sparse data

Lena Mertens[1], Matthias Sonnleitner[1], Jonathan Leach[2], Megan Agnew[2], Miles J. Padgett[1]

[1]School of Physics and Astronomy, University of Glasgow, Glasgow. G12 8QQ, UK
[2]Department of Physics, Heriot-Watt University, Edinburgh. EH14 4AS, UK

E-mail: l.mertens.1@research.gla.ac.uk, Matthias.Sonnleitner@glasgow.ac.uk, Miles.Padgett@glasgow.ac.uk

Appendix A. Proof of Equation (2) (Log-likelihood)

Proof. Let $\mathbf{n} = (n_1, \ldots, n_N)$ be an integer array of photocounts (including dark counts) and $\mathbf{I} = (I_1, \ldots, I_N)$ the corresponding array of fractional intensities of the reconstructed image. The probability to get n_j detection events given I_j and a dark-count rate ϵ follows a Poisson distribution, given by

$$p(n_j|I_j,\epsilon) = \frac{e^{-(I_j+\epsilon)}}{n_j!}(I_j+\epsilon)^{n_j}. \tag{A.1}$$

The probability for an entire image is then a product of the probabilities for the individual measurement pixels, $p(\mathbf{n}|\mathbf{I},\epsilon) = \prod_j p(n_j|I_j,\epsilon)$.

Within our image reconstruction, we are searching for the intensity distribution $\mathbf{I}$ *given* the set of measurements $\mathbf{n}$. According to Bayes' theorem [6], the probability for $\mathbf{I}$ is given as

$$p(\mathbf{I}|\mathbf{n},\epsilon) = \frac{p(\mathbf{n}|\mathbf{I},\epsilon)\, p(\mathbf{I}|\epsilon)}{p(\mathbf{n}|\epsilon)} \tag{A.2}$$

where $p(\mathbf{I}|\epsilon)$ is the *prior* representing all our information on the intensities before considering the data. The normalisation factor $p(\mathbf{n}|\epsilon) = \int p(\mathbf{n}|\mathbf{I},\epsilon)\, p(\mathbf{I}|\epsilon)\, d\mathbf{I}$ is an N-dimensional integral.

The maximum likelihood image is given by the set of intensities maximising the probability distribution given in Equation A.2, i. e.

$$\mathbf{I}_{\mathrm{Lmax}} := \arg\max_{\mathbf{I}} p(\mathbf{I}|\mathbf{n},\epsilon). \tag{A.3}$$

Since the logarithm is a monotonic function, we have $\arg\max_{\mathbf{I}} p(\mathbf{I}|\mathbf{n},\epsilon) = \arg\max_{\mathbf{I}} Lnp(\mathbf{I}|\mathbf{n},\epsilon)$, or

$$\mathbf{I}_{\mathrm{Lmax}} = \arg\max_{\mathbf{I}} \left[Ln\{p(\mathbf{n}|\mathbf{I},\epsilon)\} + Ln\{p(\mathbf{I}|\epsilon)\}\right]. \tag{A.4}$$

We have thereby eliminated the problematic normalisation term $p(\mathbf{n}|\epsilon)$ which is independent of $\mathbf{I}$ and therefore does not contribute to our search for $\mathbf{I}_{\mathrm{Lmax}}$.

The first term in Equation (A.4) can be calculated from (A.1) and gives

$$\begin{aligned} Ln\{p(\mathbf{n}|\mathbf{I},\epsilon)\} &= Ln \prod_j \frac{e^{-(I_j+\epsilon)}}{n_j!}(I_j+\epsilon)^{n_j} \\ &= \sum_j \Big(-(I_j+\epsilon) + n_j Ln(I_j+\epsilon) - Ln(n_j!)\Big) \end{aligned} \tag{A.5}$$

which is the log likelihood function given in Equation 2. For $Ln\mathcal{L}$ used in the optimisation we only need to keep terms depending on I_j.

The term involving the prior is more tricky as our "prior knowledge" about the true image can be arbitrarily defined. In this work we assume that the second spatial derivative of the intensity at the jth pixel, $\frac{d^2}{d{x_j}^2}I_j$, follows a normal distribution of width σ centred at zero. The width σ is chosen to reflect the anticipated level of detail. If we assume that the image is very smooth then σ should be set at a small value, whereas if we think that the image has lots of fine details σ should be large.

For the prior $p(\mathbf{I}|\epsilon) \equiv p(\mathbf{I})$ from Equation (A.4) this means that

$$
\begin{aligned}
Lnp(\mathbf{I}) &= Ln \prod_j (2\pi\sigma^2)^{-1/2} e^{-\frac{1}{2\sigma^2}\left(\frac{d^2}{dx_j{}^2} I_j\right)^2} \\
&\propto -\frac{1}{2\sigma^2} \sum_j \left(\tfrac{d^2}{dx_j{}^2} I_j\right)^2
\end{aligned} \tag{A.6}
$$

where we have again discarded terms that don't depend on the reconstructed image, I_j. The last line is exactly the regularization term $-\lambda R_{TC^2}$ with R_{TC^2} defined in **Equation (5)**, and $\lambda = 1/(2\sigma^2)$.

The optimal reconstructed image is then given by

$$
\mathbf{I}_{\mathrm{Lmax}} = \arg\max_{\mathbf{I}} \sum_j \left(n_j Ln(I_j + \epsilon) - I_j\right) - \frac{1}{2\sigma^2} \sum_j \left(\tfrac{d^2}{dx_j{}^2} I_j\right)^2 . \tag{A.7}
$$

which is equivalent to the expression for our cost function, defined in Equation 3.

□

Appendix B. Proof that expected reduction in log-likelihood is 0.5 per pixel

Proof. As described in Equation (3) and Appendix A we use a combination of the log-likelihood function $Ln\mathcal{L}(\mathbf{n}, \mathbf{I})$ and a regularisation function $R(\mathbf{I})$ to reconstruct an intensity distribution $\mathbf{I}_{\mathrm{Lmax}}$. The balance between sticking to the data, represented by the log-likelihood, and the desire for smoothness, given by the regularisation, is then found by varying the parameter λ.

To find out how far we can push the reconstruction we have to know how the log-likelihood changes during that process. The optimal image we could obtain by reconstruction would obviously be the true image represented by an intensity set $\mathbf{I}_{\mathrm{t}}$. As the measured photocounts follow a Poisson distribution we can calculate the expected log-likelihood for an individual pixel with true intensity I_{t} as

$$
\mathrm{E}\left[Ln\mathcal{L}(n; I_{\mathrm{t}})\right] := \sum_{n=0}^{\infty} Ln\mathcal{L}(n; I_{\mathrm{t}})\ p(n|I_{\mathrm{t}}) = I_{\mathrm{t}}(Ln(I_{\mathrm{t}}) - 1) \tag{B.1}
$$

with $Ln\mathcal{L}(n; I_{\mathrm{t}})$ for a single pixel as given by Equation (2) and the Poisson distribution for $p(n|I)$ given in Equation (1), where we now set the dark-count rate $\epsilon = 0$ for simplicity.

We can compare this to the expected log-likelihood for the intensity we use to initialise the regularisation procedure, $I_{\mathrm{init}} = n$,

$$
\mathrm{E}\left[Ln\mathcal{L}(n; I_{\mathrm{init}})\right] := \sum_{n=0}^{\infty} Ln\mathcal{L}(n; I_{\mathrm{init}})\ p(n|I_{\mathrm{t}}) . \tag{B.2}
$$

The difference between the initial and the true log-likelihood, $\mathrm{E}\left[Ln\mathcal{L}(n; I_{\mathrm{init}})\right] - \mathrm{E}\left[Ln\mathcal{L}(n; I_{\mathrm{t}})\right]$ tells us how far we can reduce the log-likelihood per pixel during the regularisation procedure. Figure B1 gives this expected difference as a function of the true intensity I_{t}.

Of course the true intensity I_{t} at each pixel is unknown, which is why we use the bootstrapping algorithm to get an estimate on $\mathrm{E}\left[Ln\mathcal{L}(n; I_{\mathrm{init}})\right] - \mathrm{E}\left[Ln\mathcal{L}(n; I_{\mathrm{t}})\right]$ using the mock data procedure. As described in Figure 3 the algorithm then adjusts the parameter λ such that the final regularisation reaches the anticipated reduction in the log-likelihood.

In figure B1 we see that the difference in log-likelihood reaches a constant value of $1/2$ for $I_{\mathrm{t}} \gg 1$. For these high intensities we get can approximate the Poissonian distribution by a Gaussian with expectation value I and width $\sqrt{I}$, i. e. $p(n|I) \to \mathrm{G}(n; I_{\mathrm{t}}) := (2\pi I)^{-1/2} \exp\left[-(x - I)^2/(2I)\right]$ with a log-likelihood $Lnp(n|I) \to -\frac{1}{2}Ln(2\pi I) - \frac{(n-I)^2}{2I}$.

The resulting expected log-likelihoods from Equations (B.1) and (B.2) then read

$$
\mathrm{E}\left[Ln\mathcal{L}(n; I_{\mathrm{t}})\right] \to -\int_{-\infty}^{\infty} \mathrm{G}(n; I_{\mathrm{t}}) \left(\frac{1}{2} Ln(2\pi I_{\mathrm{t}}) + \frac{(n - I_{\mathrm{t}})^2}{2 I_{\mathrm{t}}}\right) \mathrm{d}n = -\frac{1}{2} Ln(2\pi I_{\mathrm{t}}) - \frac{1}{2} , \tag{B.3}
$$

$$
\mathrm{E}\left[Ln\mathcal{L}(n; I_{\mathrm{init}})\right] \to -\int_{-\infty}^{\infty} \mathrm{G}(n; I_{\mathrm{t}}) \frac{1}{2} Ln(2\pi n)\ \mathrm{d}n \approx -\frac{1}{2} Ln(2\pi I_{\mathrm{t}}) + \frac{1}{4 I_{\mathrm{t}}} , \tag{B.4}
$$

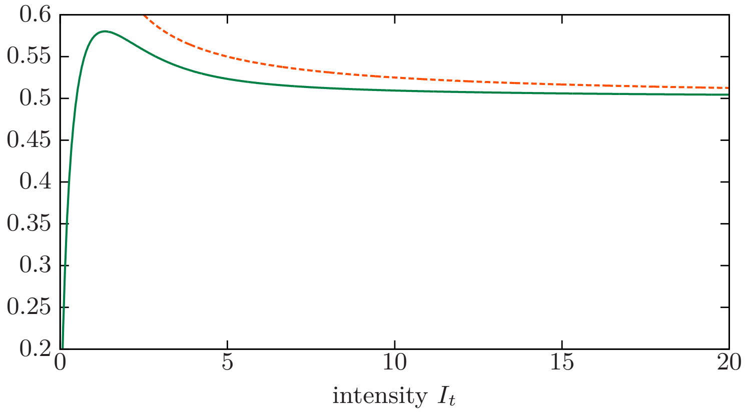

Figure B1: The expected difference between the initial and the optimal log likelihoods for an individual pixel $\mathrm{E}\left[Ln\mathcal{L}(n; I_{init})\right] - \mathrm{E}\left[Ln\mathcal{L}(n; I_t)\right]$ *as given (respectively) by Equations* (B.2) *and* (B.1) *as function of the true intensity* I_t *(green solid curve). The dashed orange curve gives the approximation using Gaussian noise valid for high intensities given in Equation* (B.5).

where we expanded $Ln(2\pi n) \approx Ln(2\pi I_\mathrm{t}) + \frac{n-I_\mathrm{t}}{I_\mathrm{t}} - \frac{(n-I_\mathrm{t})^2}{2I_\mathrm{t}^2}$ to approximate the final integral. We thus see that

$$\mathrm{E}\left[Ln\mathcal{L}(n; I_\mathrm{init})\right] - \mathrm{E}\left[Ln\mathcal{L}(n; I_\mathrm{t})\right] \to \frac{1}{4I_\mathrm{t}} + \frac{1}{2} \approx \frac{1}{2} \tag{B.5}$$

for $I_\mathrm{t} \gg 1$. This explains the constant difference between the log-likelihood for the optimal image ($I = I_\mathrm{t}$) and the initial guess ($I = n$) for $I_\mathrm{t} \gg 1$. □
